# Adhesive Intestinal Obstruction Caused by Extragastrointestinal Anisakiasis

**DOI:** 10.4269/ajtmh.14-0673

**Published:** 2015-04-01

**Authors:** Yasuyuki Takamizawa, Yoshifumi Kobayashi

**Affiliations:** Department of Surgery, Suwa Central Hospital, Nagano, Japan

**Figure 1.**
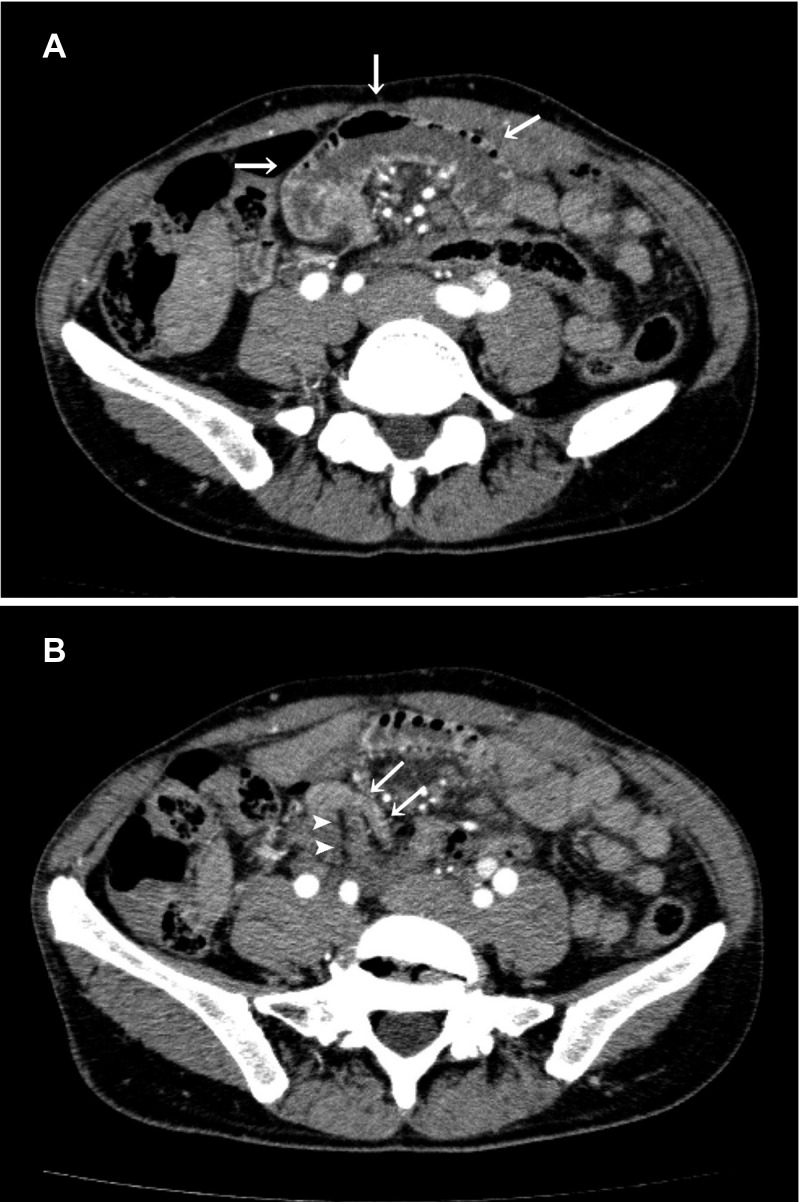
(**A**) Contrast-enhanced computed tomography scan of the abdomen showing a dilated upper intestine (arrows). (**B**) A band near the stenosis (arrowheads) and a collapsed intestine (arrows).

**Figure 2.**
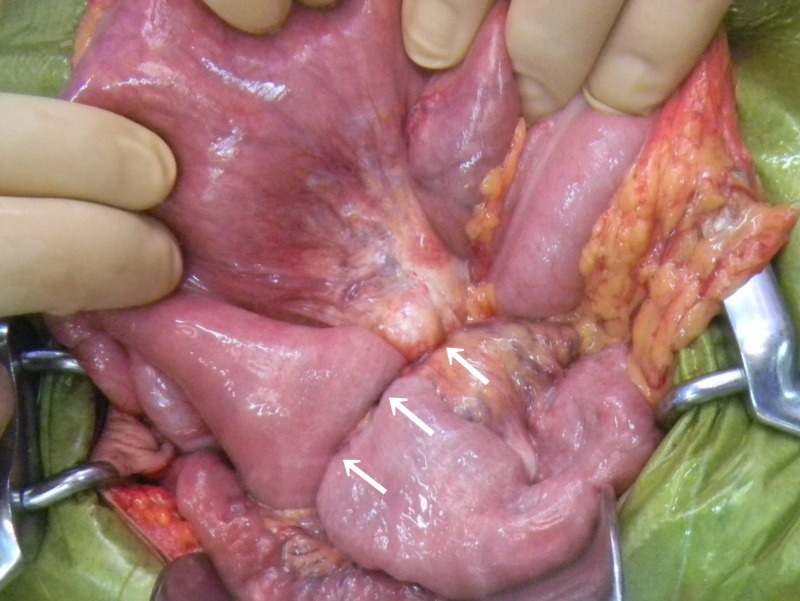
A cyclic structure (arrows) constricting the upper small intestine.

**Figure 3.**
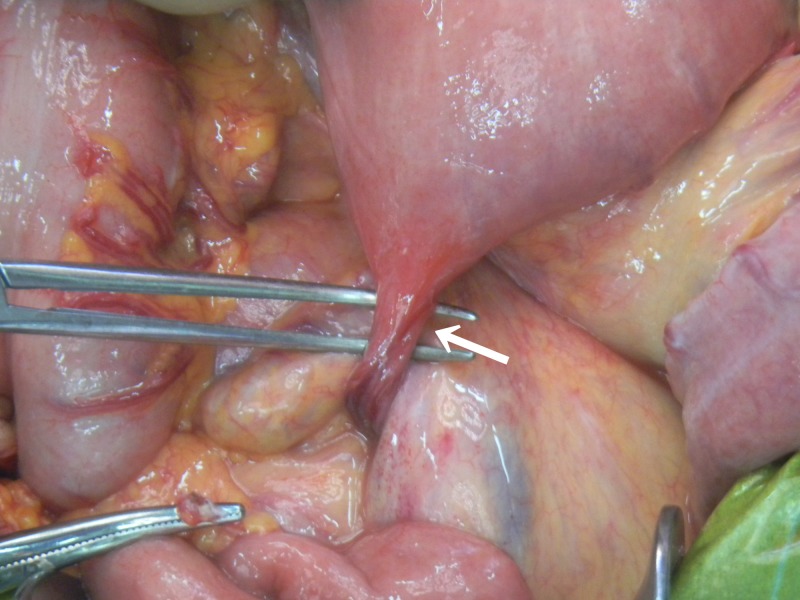
A band (arrows) protruded from the jejunum, was 10 cm distal to the ligament of Treitz, and adhered to the mesentery.

**Figure 4.**
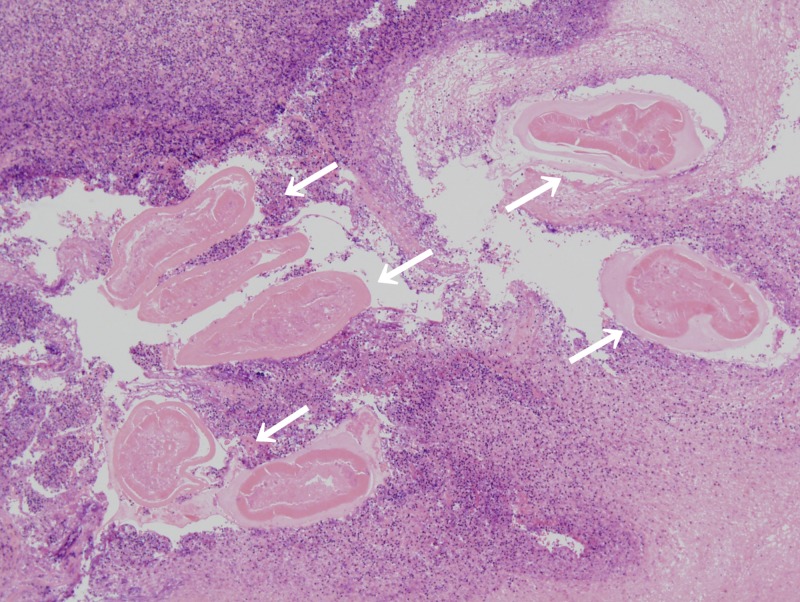
Histopathologic examination indicated an abscess surrounded by granulation tissue and a degenerated worm (arrows) inside (hematoxylin and eosin stain, ×40).

A 44-year-old Japanese woman with no history of laparotomy presented with intermittent abdominal pain that had begun 10 hours earlier. Epigastric tenderness with no muscle guarding was observed. Abdominal contrast-enhanced computed tomography showed a dilated upper small intestine and a band near the stenosis (Figure 1). Internal hernia was diagnosed, and conservative treatment with analgesics was administered. However, abdominal pain persisted. Emergency laparoscopy performed 20 hours after onset revealed two bands. Band A was a cyclic structure constricting the upper small intestine (Figure 2). Band B protruded from the jejunum, was 10 cm distal to the ligament of Treitz, and adhered to the mesentery (Figure 3). Both bands, which were comprised of fibrous tissue, were surgically excised. Band B contained an abscess surrounded by granulation tissue and a degenerated worm inside (Figure 4). Immunostaining revealed the worm to be *Anisakis simplex*. Thus, extragastrointestinal anisakiasis may have caused adhesive intestinal obstruction. Subsequently, the patient recovered completely.

Anisakiasis occurs after eating raw fish or cephalopods contaminated by larvae of the Anisakidae family. Most *Anisakis* spp. parasitize the gastrointestinal tract, usually the stomach.^1^ Rarely, anisakiasis occurs ectopically outside the gastrointestinal tract while being asymptomatic and granulomatous.^2^ Extragastrointestinal anisakiasis should be considered as a possible cause of adhesive intestinal obstruction.
